# P12 Post-streptococcal reactive arthritis and panniculitis in pregnancy: a case report

**DOI:** 10.1093/rap/rkae117.043

**Published:** 2024-11-05

**Authors:** Shumaila Baloch

**Affiliations:** Warwick Hospital, Warwick, United Kingdom

## Abstract

**Introduction:**

Reactive arthritis is an inflammatory condition that can occur following an infection, commonly caused by bacterial pathogens such as Streptococcus. Pregnancy introduces unique challenges in managing autoimmune and inflammatory conditions due to the need to balance maternal and foetal health. This case report highlights the diagnosis and management of post-streptococcal reactive arthritis and panniculitis in a 16-week pregnant patient, focusing on the use of steroids and the resulting impact on her gestational diabetes

**Case description:**

A 25-year-old woman, 16 weeks pregnant with no known comorbidities, presented with bilateral ankle pain and swelling for the last two weeks, preceded by an episode of tonsillitis. Initially, the pain was localised to her right ankle and left wrist, then involved her left ankle. She was treated with antibiotics for presumed cellulitis, and a doppler ultrasound ruled out deep vein thrombosis. Examination revealed swollen, inflamed ankles with limited range of motion due to pain. The rest of the examination was unremarkable. This was her second pregnancy and had no complications in her first pregnancy.

Routine investigations were done; her C-reactive protein (CRP) was 126 and erythrocyte sedimentation rate (ESR) was 56, with a normal white cell count. Autoimmune workup, including rheumatoid factor (RF), antinuclear antibodies (ANA), and HLA-B27, was negative, but her anti-streptolysin O (ASO) titer was borderline positive. She was diagnosed with post-streptococcal reactive arthritis. After discussing the risks and benefits of NSAID use in the second trimester, we started her on prednisolone 20 mg with a tapering plan.

The patient showed clinical improvement, but upon tapering the steroids to 5 mg daily, she developed well-demarcated erythema with desquamation and epidermal changes over both ankles. The left ankle was tender on the medial side, but the right was non-tender. Her angiotensin-converting enzyme (ACE) levels were negative, and calcium levels were normal. Her steroid dose was increased again to 20 mg with a slow tapering plan.

She also developed gestational diabetes mellitus and was started on insulin by the diabetes team. Her foetal scan was reassuring and was under obstetrician.

A dermatology team reviewed her and suggested the rash was likely panniculitis, which had improved significantly with steroids, so a biopsy was not performed. With careful management, her symptoms resolved, and she successfully discontinued steroid therapy as planned.

**Discussion:**

This case highlights the complex nature of managing post-streptococcal reactive arthritis and panniculitis in a pregnant patient. The initial presentation with a sore throat and subsequent development of arthritis and panniculitis, along with raised inflammatory markers and a positive ASO titer, strongly suggested reactive arthritis triggered by a recent streptococcal infection. Given the foetal risk from NSAIDs, steroid therapy was chosen for its safety profile in pregnancy. While steroid therapy provided symptomatic relief, it led to gestational diabetes, requiring careful monitoring and adjustment of the patient’s diabetic management.

The emergence of panniculitis during steroid tapering added another layer of complexity, necessitating collaboration with dermatology specialists for a revised approach. The dermatological team agreed with the recommendation of slow tapering of steroids, which were crucial in managing this complication. This case highlights the importance of a multidisciplinary approach and close monitoring to ensure optimal outcomes for both the mother and the developing foetus.

**Key learning points:**

• The diagnosis of post-streptococcal reactive arthritis should be considered in pregnant patients presenting with joint inflammation and a recent history of infection. Prompt recognition and appropriate treatment are essential to prevent long-term joint damage.

• Careful monitoring of glycaemic control is crucial when initiating steroid therapy in pregnant patients, as it may lead to uncontrolled diabetes and can cause foetal problems. Close collaboration between obstetric and endocrine specialists is necessary to optimise diabetes management while minimising foetal risks.

• Collaboration with dermatology specialists can aid in the management of complications such as panniculitis during steroid tapering in pregnant patients. A multidisciplinary approach ensures comprehensive care and improves outcomes for both the mother and the developing foetus.

